# Immunogenicity and safety of a quadrivalent inactivated influenza vaccine in pregnant women: a randomized, observer-blind trial

**DOI:** 10.1080/21645515.2019.1667202

**Published:** 2019-10-07

**Authors:** Timo Vesikari, Miia Virta, Seppo Heinonen, Cécile Eymin, Nathalie Lavis, Anne Laure Chabanon, Viviane Gresset-Bourgeois

**Affiliations:** a Vaccine Research Center, Tampere University, Tampere, Finland; b Tampere Vaccine Research Clinic, Vaccine Research Center, Tampere University, Tampere, Finland; c Department of Obstetrics and Gynecology, Helsinki University Hospital, Helsinki, Finland; d University of Helsinki, Helsinki, Finland; e Medical Operations, Sanofi Pasteur, Campus Sanofi Lyon, Lyon, France; f Global Pharmacovigilance, Sanofi Pasteur, Lyon, France

**Keywords:** Immunization, influenza vaccines, newborn infant, pregnancy, safety, seasonal influenza, transplacental antibody transfer, vaccination

## Abstract

Vaccination against influenza during pregnancy provides direct protection to pregnant women and indirect protection to their infants. Trivalent inactivated influenza vaccines (IIV3s) are safe and effective during pregnancy, but quadrivalent inactivated influenza vaccines (IIV4s) have not been evaluated in pregnant women and their infants. Here, we report the results of a randomized phase IV study to evaluate the immunogenicity and safety of IIV4 vs. IIV3 in pregnant women. Participants aged ≥18 years at weeks 20 to 32 of gestation were randomly assigned in a 2:1 ratio to receive a single dose of IIV4 (n = 230) or IIV3 (n = 116). Between baseline and 21 days after vaccination, hemagglutination inhibition (HAI) antibody titers increased in both groups by similar magnitudes for the two influenza A strains and single B strain common to IIV4 and IIV3. For the additional B strain in IIV4, HAI titers were higher in IIV4 recipients than IIV3 recipients (post-/pre-vaccination geometric mean titer ratio, 6.3 [95% CI: 5.1 − 7.7] vs. 3.4 [95% CI: 2.7 − 4.3]). At delivery, in both groups, HAI antibody titers for all strains were 1.5 − 1.9-fold higher in umbilical cord blood than in maternal blood, confirming active transplacental antibody transfer. Rates of solicited and unsolicited vaccine-related adverse events in mothers were similar between the two groups. Live births were reported for all participants and there were no vaccine-related adverse events in newborns. These results suggest IIV4 is as safe and immunogenic as IIV3 in pregnant women, and that maternal immunization with IIV4 should protect newborns against influenza via passively acquired antibodies.

## Introduction

Pregnant women and young infants are among the population subgroups at greatest risk of severe illness, complications, and death from influenza.^1,2^ Hospital admissions for influenza illness are more frequent among pregnant women than non-pregnant women,^3,4^ and influenza in young infants frequently leads to hospitalization,^5^ bacterial co-infections,^6,7^ and a higher mortality rate than in older children.^8^ Severe influenza illness during pregnancy has also been associated with preterm birth and fetal death in the 2009 influenza A/H1N1 pandemic.^9^

Maternal vaccination during pregnancy is considered the most effective strategy to protect pregnant women and newborn infants against influenza.^1^ The World Health Organization (WHO)^1^ and other advisory bodies^10–12^ recommend that pregnant women are prioritized for influenza vaccination, since this provides both direct protection to pregnant women and indirect protection to their infants via transplacental maternal antibody transfer.^2,13–16^ This strategy is especially important for preventing influenza illness in infants aged less than 6 months because influenza vaccines are not licensed for use in this age group.

Inactivated influenza vaccines have been shown to be safe, immunogenic, and effective during pregnancy in several randomized controlled studies.^13–15,17^ Importantly, vaccination was shown to reduce the incidence of laboratory-confirmed influenza cases in pregnant women and their newborns by around 50%.^13–16,18,19^ Studies have also identified reduced rates of influenza-related hospitalization among infants born to women vaccinated against influenza during pregnancy.^19–21^ However, in many countries, few pregnant women receive influenza vaccines, often due to low awareness of the risks posed by the disease and concerns about the safety and efficacy of influenza vaccination during pregnancy.^22^

Until recently, most influenza vaccines have been trivalent, containing antigens from two influenza A subtypes (H1N1 and H3N2) and one influenza B lineage virus.^1,23^ However, since the 1980s, two antigenically distinct influenza B lineages have co-circulated globally, which has complicated the selection of the correct B lineage for each new influenza season.^24^ Global data indicate that differences frequently occur between the trivalent vaccine and the predominant circulating B lineage strains, which has resulted in suboptimal protection in several previous influenza seasons.^25–27^ To help ensure protection against influenza B, quadrivalent inactivated influenza vaccines (IIV4s), which contain the two A strains and a strain from each B lineage, have been developed and are generally replacing trivalent inactivated influenza vaccines (IIV3s) worldwide.^24^ Although there are no specific data on influenza B infection in pregnant women, influenza B is known to cause similar morbidity to influenza A across different populations and age groups.^28^ Vaccination during pregnancy with IIV4 instead of IIV3 would therefore extend protection to cover both circulating B lineages in pregnant women and newborns.

Whereas vaccination during pregnancy with IIV3s has been shown safe, effective, and to result in transplacental maternal antibody transfer,^13–15^ IIV4s have not been evaluated in pregnant women and their infants. Here, we report the immunogenicity and safety of an IIV4 in pregnant women, as well as birth outcomes and transplacental transfer of vaccine-induced antibodies.

## Patients and methods

### Study design

This was a Phase IV, randomized, blind observer, controlled, multi-center study conducted in pregnant women in Finland between September, 2017 and June, 2018 (EudraCT number: 2016-004763-40).

During the study enrollment period, the National Immunization Program of Finland offered vaccination with IIV3 free of charge for pregnant women at any stage of pregnancy.^29^ This led to a lower than expected rate of enrollment and, consequently, the required study power was not achieved for the original primary (non-inferiority of IIV4 immune responses vs. IIV3) and secondary (superiority of IIV4 immune response against the additional B strain) immunogenicity objectives. The study protocol was amended by replacing the immunogenicity objectives by a description of the immune response.

The revised primary objectives of the study were (a) to describe the immune response and (b) to describe the safety of one dose of IIV4 (VaxigripTetra, Sanofi Pasteur) or IIV3 (Vaxigrip, Sanofi Pasteur) 21 days after vaccination in pregnant women. Secondary objectives were to evaluate the transplacental transfer of antibody from mother to newborn from the cord blood after one dose of IIV4 or IIV3, and to evaluate the safety profile of IIV4 and IIV3 in pregnant women and in terms of birth outcome. The study was approved by the national ethics committee and conducted in accordance with the Declaration of Helsinki, Good Epidemiological Practice, and national regulations. All participants provided their written informed consent before inclusion.

### Participants

The study included pregnant women aged ≥18 years at 20 to 32 weeks of pregnancy. This gestational age was chosen to assess transplacental antibody transfer at birth. Potential subjects were excluded if they had received any vaccine within 4 weeks before study vaccination; already been vaccinated against influenza in the 2017 − 2018 season; experienced pregnancy complications during the current pregnancy; or had a chronic illness that, in the opinion of the investigator, might interfere with the study assessments. Further exclusion criteria are provided in the **Supplementary Materials**.

### Vaccines

The study used the WHO-recommended 2017–2018 Northern Hemisphere formulations of IIV4 and IIV3. Both vaccines were inactivated, split-virion, thimerosal-free, and were provided in pre-filled syringes of 0.5 ml. IIV3 contained 15 µg hemagglutinin per strain of A/Michigan/45/2015 (H1N1)pdm09-like virus, A/Hong Kong/4801/2014 (H3N2)-like virus, and B/Brisbane/60/2008-like virus (B/Victoria lineage). IIV4 contained 15 µg hemagglutinin of each of the above strains plus 15 µg hemagglutinin of B/Phuket/3073/2013-like virus (B/Yamagata lineage).

### Study conduct

Participants were randomly assigned in a 2:1 ratio to receive a single dose (0.5 ml) of IIV4 or IIV3 by intramuscular injection. Randomization was performed using the permuted block method and scratchable randomization lists were used at each site to communicate which vaccine was to be injected. The study was conducted in a blind observer manner, where neither the participants nor the investigators responsible for safety assessment knew which vaccine was administered. Blood samples were taken from the study participants at baseline (day 0) and 21 days after vaccination to determine hemagglutination inhibition (HAI) antibody titers. To study the transfer of maternal antibodies, blood samples were also taken from the participants within 4 days of delivery and from the umbilical cord on the day of delivery.

### Immunogenicity assessments

Anti-hemagglutinin antibody levels were measured in the participants before vaccination (day 0) and 21 days after the last vaccination, and in the participants and umbilical cord blood after delivery, by HAI assay as described previously.^30^ Geometric mean titers (GMTs), the geometric mean of the individual ratios of post-vaccination vs. pre-vaccination (day 0) titers, percentages of individuals with titers ≥1:40, and seroconversion or significant increase in HAI titer were calculated. Seroconversion was defined as a pre-vaccination HAI titer <1:10 and a post-vaccination titer ≥1:40; and a significant increase as a pre-vaccination HAI titer ≥1:10 and ≥4-fold increase in post- vs. pre-vaccination HAI titer.

### Safety assessments

Safety was assessed according to International Conference on Harmonization guidelines.^31^ Immediate unsolicited adverse events (AEs) were defined as those occurring within 30 min following vaccination. Subjects recorded information about solicited reactions in a diary card for up to 7 days after vaccination, and about unsolicited AEs up to 21 days after vaccination. Any serious AEs were reported to investigators throughout the study, from inclusion until delivery. The newborn baby underwent a physical examination after delivery. Any complications during pregnancy or delivery were considered as AEs, and in some cases were considered serious AEs (e.g., spontaneous abortions, fetal death, stillbirth, and congenital anomalies reported in the baby). Investigators assessed unsolicited AEs and serious AEs as unrelated or possibly related to the vaccination. AEs were coded with Medical Dictionary for Regulatory Activities (MedDRA) terminology (version 19.0).

### Sample size

Under the original trial protocol, with 486 subjects planned for enrollment in the IIV4 group and 243 in the IIV3 group, the study was planned to have an overall power of >80% for the original non-inferiority and superiority immunogenicity endpoint analyses. Following the low rate of enrollment, the study protocol was amended to make the immunogenicity objectives descriptive only, and so recalculating the sample size was not required.

### Statistical analysis

All analyses were descriptive. 95% confidence intervals (CIs) were calculated using SAS® version 9.4 (SAS Institute, Cary, NC, USA). Missing or incomplete data were not replaced, with the exception that all HAI titers under the lower limit of quantitation (10) were assigned a value of 5 and all HAI titers above the upper limit of quantitation (10,240) were assigned a value of 10,240. Immunogenicity was analyzed in all eligible subjects who were vaccinated according to protocol and who had valid serology results. Safety was analyzed in all subjects who received a study vaccine.

## Results

### Study population

The study enrolled 346 subjects in Finland between September 15, 2017 and January 26, 2018. Enrollment was stopped on January 26, 2018, before reaching the number of subjects to achieve the study power for the original primary (non-inferiority of IIV4 immune responses vs. IIV3) and secondary (superiority of IIV4 immune response against the additional B strain) immunogenicity objectives. This was due to an unforeseen increase in free-of-charge IIV3 vaccination among pregnant women under the Finnish influenza vaccination program.

**Figure 1.**
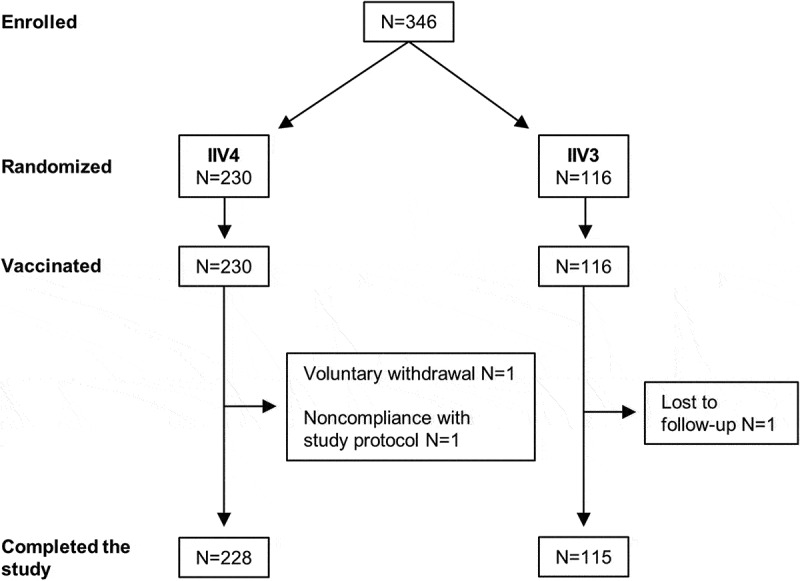
Study design and disposition of participants. Pregnant women aged ≥18 years at 20 to 32 weeks of pregnancy were randomly assigned 2:1 to receive IIV4 or IIV3 by intramuscular injection.

By the end of the enrollment period, 230 subjects had been randomized to receive IIV4 and 116 had been randomized to receive IIV3 (Figure 1). The study ended on June 14, 2018 and was completed by all subjects except for two vaccinated with IIV4 (one voluntarily withdrawal and one due to noncompliance with the study protocol [missing visit] because of pre-term delivery, which was considered unrelated to the vaccine) and one vaccinated with IIV3 (lost to follow-up).

Subject demographics were similar between the two groups: the median age was 32.1 years (range: 20.3 − 44.3 years) for the women vaccinated with IIV4 and 30.7 years (range: 20.5 − 42.5 years) for those vaccinated with IIV3, and the median gestational age at enrollment was 25 weeks (interquartile range [IQR]: 22 − 29 weeks) for the IIV4 recipients and 24 weeks (IQR: 21 − 27 weeks) for the IIV3 recipients.

### Immunogenicity

Baseline HAI antibody titers for all vaccine strains were similar between the pregnant women vaccinated with IIV4 and those vaccinated with IIV3 (Table 1). At 21 days after vaccination, HAI antibody titers had increased in IIV4 recipients for all vaccine strains by 3.8-fold to 8.6-fold from baseline titers. The increase in HAI antibody titers was similar in IIV3 recipients for the A/H1N1, A/H3N2, and B/Victoria strains common to both vaccines (post-/pre-vaccination GMT ratios: 5.3 − 9.6). However, IIV4 HAI titers were higher than IIV3 titers for the B/Yamagata strain (GMT ratios: 6.3 [95% CI: 5.1 − 7.7] vs. 3.4 [95% CI: 2.7 − 4.3]). At least 95.8% of IIV4 recipients and 94.5% of IIV3 recipients had titers ≥1:40 against individual vaccine strains at 21 days after vaccination. Rates of seroconversion/significant increase in titers were 38% to 61% in IIV4 recipients and 41% to 62% in IIV3 recipients for the three common vaccine strains. For B/Yamagata, more participants vaccinated with IIV4 seroconverted or had significant increases in titers than those vaccinated with IIV3 (59.7% [95% CI: 52.9 − 66.3%] vs. 38.5% [95% CI: 29.4 − 48.3%]).

**Table 1.** HAI antibody responses.

		A/H1N1		A/H3N2		B/Victoria		B/Yamagata	
Measure	Day	IIV4	IIV3	IIV4	IIV3	IIV4	IIV3	IIV4	IIV3^a^
N	-	216	109	216	109	216	109	216	109
HAI GMT (95% CI)	0	138 (114 − 166)	121 (88.4 − 166)	39.6 (32.2 − 48.6)	40.0 (29.4 − 54.5)	67.1 (55.2 − 81.4)	72.5 (54.7 − 96.1)	159 (131 − 193)	155 (120 − 202)
	21	525 (466 − 592)	638 (529 − 769)	341 (286 − 407)	369 (283 − 483)	568 (496 − 651)	697 (569 − 855)	993 (870 − 1134)	529 (415 − 674)
HAI titer ≥1:40, % (95% CI)	0	86.1 (80.8 − 90.4)	78.0 (69.0 − 85.4)	55.1 (48.2 − 61.8)	53.2 (43.4 − 62.8)	69.4 (62.8 − 75.5)	65.1 (55.4 − 74.0)	85.2 (79.7 − 89.6)	83.5 (75.2 − 89.9)
	21	99.5 (97.4 − 100)	100 (96.7 − 100)	95.8 (92.2 − 98.1)	94.5 (88.4 − 98.0)	100 (98.3 − 100)	99.1 (95.0 − 100)	100 (98.3 − 100)	97.2 (92.2 − 99.4)
GMT ratio^b^ (95% CI)	21/0	3.8 (3.1 − 4.7)	5.3 (3.7 − 7.6)	8.6 (6.9 − 10.9)	9.2 (6.6 − 13.0)	8.5 (6.8 − 10.6)	9.6 (6.9 − 13.4)	6.3 (5.1 − 7.7)	3.4 (2.7 − 4.3)
Seroconversion or significant increase^c^, % (95% CI)	21/0	38.0 (31.5 − 44.8)	41.3 (31.9 − 51.1)	59.3 (52.4 − 65.9)	62.4 (52.6 − 71.5)	61.1 (54.3 − 67.7)	60.6 (50.7 − 69.8)	59.7 (52.9 − 66.3)	38.5 (29.4 − 48.3)

Values are for all subjects completing the study according to protocol. IIV3 contained 15 µg hemagglutinin per strain of A/Michigan/45/2015 (H1N1)pdm09-like virus, A/Hong Kong/4801/2014 (H3N2)-like virus, and B/Brisbane/60/2008-like virus (B/Victoria lineage). IIV4 contained 15 µg hemagglutinin of each of the above strains plus 15 µg hemagglutinin of B/Phuket/3073/2013-like virus (B/Yamagata lineage). Abbreviations: CI, confidence interval; GMT, geometric mean titer; HAI, hemagglutination inhibition; IIV4, quadrivalent inactivated influenza vaccine; IIV3, trivalent inactivated influenza vaccine

^a^ The IIV3 formulation did not include the B/Phuket/3073/2013-like virus (B/Yamagata strain)

^b^ Geometric mean of the individual ratios of the post-vaccination (day 21) HAI titer divided by the pre-vaccination (day 0) HAI titer

^c^ Seroconversion was defined as a pre-vaccination (day 0) HAI titer <1:10 and a post-vaccination (day 21) HAI titer ≥1:40, and a significant increase was defined as a pre-vaccination HAI titer ≥1:10 and a ≥ 4-fold increase in HAI titer

**Figure 2.**
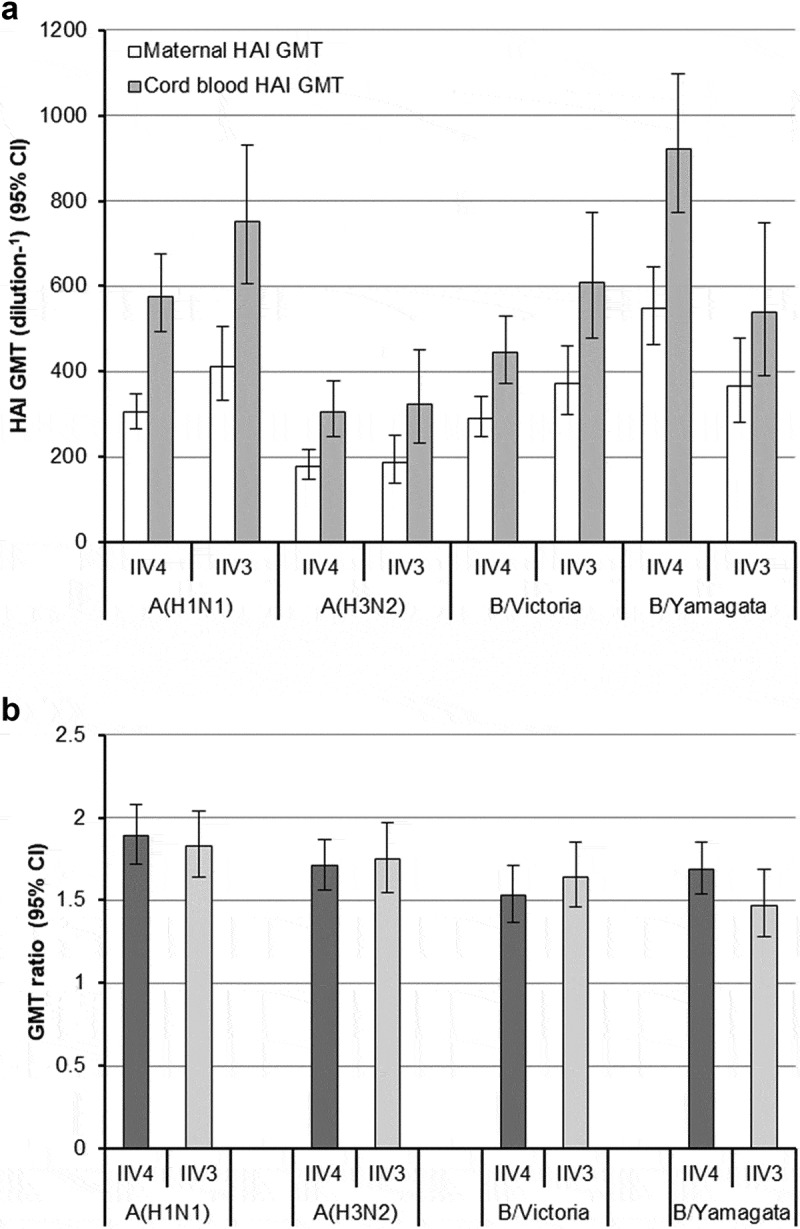
Maternal and cord blood hemagglutination inhibition (HAI) antibody titers at delivery. (a) Maternal and cord blood HAI geometric mean titers (GMTs). (b) GMT ratio: the geometric mean of the individual ratios of the cord blood HAI titer divided by the maternal HAI titer at delivery. The trivalent inactivated influenza vaccine (IIV3) contained A/H1N1, A/H3N2, and a strain from the B/Victoria lineage. The quadrivalent inactivated influenza vaccine (IIV4) contained each of the above strains plus a strain from the B/Yamagata lineage. Values are for all subjects completing the study according to protocol who provided blood and cord blood samples at delivery. For GMT ratios (b), mothers that had twins were counted twice. Abbreviation: CI, confidence interval.

At delivery, HAI antibody titers for the A/H1N1 strain were almost twice as high in the umbilical cord blood as in the maternal blood in both groups (1.9-fold higher in the IIV4 group, 1.8-fold higher in the IIV3 group) and were between 1.5 and 1.7 times as high for the A/H3N2 strain and two B-lineage strains (Figure 2). The cord blood to maternal blood HAI GMT ratios were similar between the IIV4 and IIV3 groups for all four vaccine strains.

### Safety

The proportions of subjects reporting solicited reactions and unsolicited vaccine-related AEs were similar between the two vaccine groups (Table 2). Injection site reactions were reported by most subjects vaccinated with IIV4 (90.0%) and IIV3 (80.9%). These were typically mild (grade 1) in intensity and resolved within 3 days. Pain at the injection site was more frequently reported by subjects vaccinated with IIV4 than those vaccinated with IIV3 (88.7% [95% CI: 83.9 − 92.5%] vs. 76.5% [95% CI: 67.7 − 83.9%]) (Supplemental Table 1). The most frequently reported solicited systemic reaction in the two vaccine groups was headache. The most common vaccine-related unsolicited AEs were injection site pruritus, fatigue, and oropharyngeal pain (Supplemental Table 1). None of the subjects reported grade 3 (severe) solicited reactions or unsolicited vaccine-related AEs.

**Table 2.** Proportions of subjects experiencing adverse events within 21 days after vaccination.

	IIV4		IIV3	
	(N = 230)		(N = 116)	
Event	n	% (95% CI)	n	% (95% CI)
Immediate unsolicited AE	0	0.0 (0.0 − 1.6)	0	0.0 (0.0 − 3.1)
Solicited reaction	214	93.0 (88.9 − 96.0)	106	92.2 (85.7 − 96.4)
Solicited injection site reaction	207	90.0 (85.4 − 93.6)	93	80.9 (72.5 − 87.6)
Solicited systemic reaction	155	67.4 (60.9 − 73.4)	83	72.2 (63.0 − 80.1)
Unsolicited AE	127	55.2 (48.5 − 61.8)	61	52.6 (43.1 − 61.9)
Vaccine-related	29	12.6 (8.6 − 17.6)	17	14.7 (8.8 − 22.4)
Non-serious	126	54.8 (48.1 − 61.3)	61	52.6 (43.1 − 61.9)
Non-serious vaccine-related	29	12.6 (8.6 − 17.6)	17	14.7 (8.8 − 22.4)
Injection site non-serious vaccine-related	9	3.9 (1.8 − 7.3)	4	3.4 (0.9 − 8.6)
Systemic non-serious vaccine‑related	23	10.0 (6.4 − 14.6)	13	11.2 (6.1−18.4)
AE leading to study discontinuation	0	0.0 (0.0 − 1.6)	0	0.0 (0.0 − 3.1)
Serious AE	2	0.9 (0.1 − 3.1)	0	0.0 (0.0 − 3.1)
Vaccine-related	0	0.0 (0.0 − 1.6)	0	0.0 (0.0 − 3.1)
Death	0	0.0 (0.0 − 1.6)	0	0.0 (0.0 − 3.1)

Abbreviations: AE, adverse event; CI, confidence interval; IIV4, quadrivalent inactivated influenza vaccine; IIV3, trivalent inactivated influenza vaccine

Two serious AEs were experienced within 21 days of vaccination (one hospitalization for pneumonia and one hospitalization for pre-eclampsia), although neither was considered vaccine-related. No immediate unsolicited AEs were reported and none of the participants had AEs leading to discontinuation from the study.

### Birth outcomes

All birth outcomes reported were live births. One subject vaccinated with IIV4 and three subjects vaccinated with IIV3 gave birth to twins. Baby birth weights and heights, and the proportion of participants having vaginal and C-section deliveries were also similar between the two groups.

Six congenital abnormalities were reported in babies of IIV4-vaccinated mothers (2.6%) and five in babies of IIV3-vaccinated mothers (4.2%), most of which were hip dysplasias. There were no vaccine-related adverse events in newborns.

## Discussion

By including strains from both circulating B lineages, IIV4s extend the protection provided by IIV3s against influenza. However, the safety and efficacy of IIV4s in pregnant women – a group prioritized for vaccination^1^ – has not been previously evaluated. The results from this randomized study showed that IIV4 had similar immunogenicity to IIV3 and was well tolerated in pregnant women vaccinated during the second or third trimester of pregnancy. In addition, high HAI antibody titers were found in the cord blood after delivery, suggesting maternal immunization conferred protection to newborns via transplacental antibody transfer.

Consistent with studies in non-pregnant adults,^30,32,33^ IIV4 induced similar HAI antibody titers to IIV3 in pregnant women for the three common vaccine strains, and higher HAI titers for the additional B lineage strain. Baseline HAI antibody titers for A/H1N1 and B/Yamagata were high in the study population. The high baseline A/H1N1 titers might be explained by previous vaccination with the 2009 adjuvanted pandemic A/H1N1 vaccine, which was received by more than one-half of Finland’s population.^34^ The high baseline B/Yamagata titers could be related to the high circulation of this lineage in 2017 − 2018,^27^ and could have limited the difference in titer increase for this strain between IIV4 and IIV3. Nonetheless, more than 95% of IIV4 recipients had titers of 1:40 or higher against each individual vaccine strain on day 21.

As reported previously,^35^ cord blood HAI antibody titers were higher than those from maternal blood, which indicates the newborns should have been passively immunized against the influenza strains of each vaccine. In previous clinical studies, newborns of IIV3 recipients had higher HAI titers for all vaccine strains, and were more likely to have titers of 1:40 or higher, than did newborns of placebo or control vaccine recipients.^13,15^ Compared with IIV3 recipients, IIV4 recipients did not have clearly increased cord blood to maternal blood HAI GMT ratios for the additional B/Yamagata strain, perhaps again due to high baseline B/Yamagata HAI titers in the population.

The pregnant women vaccinated with IIV4 experienced no differences in solicited local and systemic reactions, or unsolicited vaccine-related AEs to those vaccinated with IIV3. The frequently observed injection site reactions, headache, and myalgia after vaccination are expected reactions from influenza vaccines, and have been reported in similar proportions of participants in other studies.^13–15,30^ Solicited injection site reactions were more frequently reported in IIV4 recipients than in IIV3 recipients (90% vs. 81%), though this slight difference was not considered medically significant. Moreover, nearly all of the solicited reactions were mild in intensity and short-lived.

None of the reported pregnancy complications or congenital abnormalities were considered vaccine-related. This is consistent with the large body of evidence affirming no increased risk of adverse pregnancy outcomes following influenza vaccination during pregnancy.^9,36,37^ The observed incidence of hip dysplasias (17.1 per 1000) was within the expected incidence rate for Scandinavian countries (0.9 − 28 per 1000).^38^

Our study is the first to evaluate the immunogenicity and safety of IIV4 specifically in pregnant women. The study was strengthened by its randomized design and by comparing to the IIV3 formulation, which has been shown safe and effective during pregnancy.^13–15^ However, due to low enrollment, our study could only report descriptive analyses. The originally planned, larger sample size should not have considerably increased the rates of observed rare serious vaccine-related AEs, given that much larger studies of IIV4,^30^ and of IIV3 in pregnant women,^13–15^ show very low rates of these events. Our study was also limited by only including pregnant women in late second or third trimester, whereas the WHO recommends influenza vaccination at any stage of pregnancy.^1^ Nonetheless, other studies have shown IIV3 has a similar safety profile at other gestational ages.^13–15,17^ Finally, the study was conducted only in Finland. Including pregnant women from other countries might have increased the size of the study and helped confirm consistency across different regions.

Consistent with the recognized safety and effectiveness of IIV3 during pregnancy,^1,11,13–15,39^ our results support the use of IIV4 to protect pregnant women against influenza. The high cord blood HAI antibody titers also suggest maternal immunization with IIV4 would protect newborns against influenza, at least as well as IIV3,^13,15^ via passively acquired antibodies. The results from this study might encourage healthcare professionals to recommend IIV4 to pregnant women, for whom vaccination coverage is often poor due to concerns about influenza vaccine safety and efficacy during pregnancy.^22^
