# Supplementary material for: Immunogenicity and safety of a quadrivalent inactivated influenza vaccine in pregnant women: a randomized, observer-blind trial
Source: Hum Vaccin Immunother. 2019 Oct 7;16(3):623–9. doi: 10.1080/21645515.2019.1667202 (PMC7227680; doi:10.1080/21645515.2019.1667202)
Supplement: Supplemental Material [file khvi-16-03-1667202-s001.docx]

# Supplemental materials for: “Immunogenicity and safety of a quadrivalent inactivated influenza vaccine in pregnant women: a randomized, observer-blind trial”

## Exclusion criteria

Potential subjects were excluded if they had:

- received any vaccine in the 4 weeks preceding study vaccination or planned to receive any vaccine in the 4 weeks following study vaccination;
- been already vaccinated against influenza with the 2017-2018 Northern Hemisphere formulation;
- received in the preceding 3 months (or were scheduled to receive) immune globulins (with the exception of Rho D immune globulin), blood, or blood-derived products;
- received immunosuppressive therapy within the preceding 6 months;
- received long-term systemic corticosteroid therapy (prednisone or equivalent for >2 consecutive weeks) within the past 3 months;
- received corticosteroids for preterm labor ≤14 days before enrollment;
- had pregnancy complications during the current pregnancy;
- known hypersensitivity or history of a life-threatening reaction to the study vaccines or their components;
- known or suspected congenital or acquired immunodeficiency;
- had thrombocytopenia, a bleeding disorder, or had received anticoagulants in the 3 weeks before the study;
- had a chronic illness that, in the opinion of the investigator, might interfere with the study assessments;
- had moderate or severe acute illness or infection on the day of vaccination or febrile illness (temperature ≥38.0°C).

**Supplementary Table 1: Solicited reactions and vaccine-related unsolicited adverse events**

|  | **IIV4** | |  | **IIV3** | |
| --- | --- | --- | --- | --- | --- |
|  | **(N=230)** | |  | **(N=116)** | |
| **Subjects experiencing at least one:** | **n** | **% (95% CI)** |  | **n** | **% (95% CI)** |
| Solicited injection site reactions | 207 | 90.0 (85.4−93.6) |  | 93 | 80.9 (72.5−87.6) |
| Pain | 204 | 88.7 (83.9−92.5) |  | 88 | 76.5 (67.7−83.9) |
| Erythema | 24 | 10.4 (6.8−15.1) |  | 16 | 13.9 (8.2−21.6) |
| Swelling | 13 | 5.7 (3.0−9.5) |  | 6 | 5.2 (1.9−11.0) |
| Induration | 8 | 3.5 (1.5−6.7) |  | 5 | 4.3 (1.4−9.9) |
| Ecchymosis | 2 | 0.9 (0.1−3.1) |  | 2 | 1.7 (0.2−6.1) |
|  |  |  |  |  |  |
| Solicited systemic reactions | 155 | 67.4 (60.9−73.4) |  | 83 | 72.2 (63.0−80.1) |
| Fever | 0 | 0.0 (0.0−1.6) |  | 2 | 1.7 (0.2−6.1) |
| Headache | 96 | 41.7 (35.3−48.4) |  | 55 | 47.8 (38.4−57.3) |
| Malaise | 67 | 29.1 (23.3−35.5) |  | 32 | 27.8 (19.9−37.0) |
| Myalgia | 85 | 37.0 (30.7−43.5) |  | 29 | 25.2 (17.6−34.2) |
| Shivering | 61 | 26.5 (20.9−32.7) |  | 30 | 26.1 (18.3−35.1) |
|  |  |  |  |  |  |
| Vaccine-related unsolicited AEs | 29 | 12.6 (8.6−17.6) |  | 17 | 14.7 (8.8−22.4) |
| Injection site pruritus | 9 | 3.9 (1.8−7.3) |  | 2 | 1.7 (0.2−6.1) |
| Injection site rash | 1 | 0.4 (0.0−2.4) |  | 1 | 0.9 (0.0−4.7) |
| Injection site warmth | 0 | 0.0 (0.0−1.6) |  | 1 | 0.9 (0.0−4.7) |
| Malaise | 0 | 0.0 (0.0−1.6) |  | 1 | 0.9 (0.0−4.7) |
| Fatigue | 3 | 1.3 (0.3−3.8) |  | 5 | 4.3 (1.4−9.8) |
| Pain | 1 | 0.4 (0.0−2.4) |  | 0 | 0.0 (0.0−3.1) |
| Diarrhea | 1 | 0.4 (0.0−2.4) |  | 0 | 0.0 (0.0−3.1) |
| Influenza-like illness | 1 | 0.4 (0.0−2.4) |  | 0 | 0.0 (0.0−3.1) |
| Nasopharyngitis | 2 | 0.9 (0.1−3.1) |  | 0 | 0.0 (0.0−3.1) |
| Rhinitis | 3 | 1.3 (0.3−3.8) |  | 1 | 0.9 (0.0−4.7) |
| Upper respiratory tract infection | 2 | 0.9 (0.1−3.1) |  | 1 | 0.9 (0.0−4.7) |
| Herpes simplex | 1 | 0.4 (0.0−2.4) |  | 0 | 0.0 (0.0−3.1) |
| Lymphadenopathy | 0 | 0.0 (0.0−1.6) |  | 1 | 0.9 (0.0−4.7) |
| Dizziness | 1 | 0.4 (0.0−2.4) |  | 0 | 0.0 (0.0−3.1) |
| Increased blood pressure | 1 | 0.4 (0.0−2.4) |  | 0 | 0.0 (0.0−3.1) |
| Cough | 1 | 0.4 (0.0−2.4) |  | 1 | 0.9 (0.0−4.7) |
| Nasal congestion | 2 | 0.9 (0.1−3.1) |  | 1 | 0.9 (0.0−4.7) |
| Oropharyngeal pain | 4 | 1.7 (0.5−4.4) |  | 4 | 3.4 (0.9−8.6) |
| Rhinorrhea | 1 | 0.4 (0.0−2.4) |  | 0 | 0.0 (0.0−3.1) |
| Eczema | 1 | 0.4 (0.0−2.4) |  | 0 | 0.0 (0.0−3.1) |
| Erythema | 1 | 0.4 (0.0−2.4) |  | 0 | 0.0 (0.0−3.1) |
| Miliaria | 1 | 0.4 (0.0−2.4) |  | 0 | 0.0 (0.0−3.1) |
| Pruritus | 1 | 0.4 (0.0−2.4) |  | 0 | 0.0 (0.0−3.1) |

Abbreviations: AE, adverse event; CI, confidence interval; IIV4, quadrivalent inactivated influenza vaccine; IIV3, trivalent inactivated influenza vaccine
